# Supplementary material for: Development of a computer-aided design software for the quantitative evaluation of aesthetic damage
Source: PLoS One. 2019 Dec 18;14(12):e0226322. doi: 10.1371/journal.pone.0226322 (PMC6919621; doi:10.1371/journal.pone.0226322)
Supplement: S1 Code site — 1. (ZIP) [file pone.0226322.s001.zip › [EESC_jr][Projeto]Relatorio_07_21/reports/templates_zip/gentelella-master.zip!/gentelella-master/vendors/DateJS/reports/lcov-report/core/index.html]

Code coverage report for core/


# Code coverage report for core/

## Statements: 82.29% (1385 / 1683)      Branches: 70.61% (793 / 1123)      Functions: 83.48% (288 / 345)      Lines: 82.5% (1377 / 1669)      Ignored: none

All files » core/

| File |  | Statements |  | Branches |  | Functions |  | Lines |  |
| --- | --- | --- | --- | --- | --- | --- | --- | --- | --- |
| core-prototypes.js |  | 95.1% | (291 / 306) | 83.33% | (195 / 234) | 100% | (50 / 50) | 96.28% | (285 / 296) |
| core.js |  | 96.77% | (120 / 124) | 90.11% | (82 / 91) | 91.18% | (31 / 34) | 98.36% | (120 / 122) |
| extras.js |  | 61.76% | (42 / 68) | 33.33% | (15 / 45) | 92.31% | (12 / 13) | 61.76% | (42 / 68) |
| format\_parser.js |  | 96.95% | (159 / 164) | 86.13% | (118 / 137) | 94.29% | (33 / 35) | 96.95% | (159 / 164) |
| i18n.js |  | 95.45% | (147 / 154) | 75% | (69 / 92) | 94.12% | (32 / 34) | 95.45% | (147 / 154) |
| parser.js |  | 72.34% | (34 / 47) | 87.5% | (28 / 32) | 62.5% | (5 / 8) | 72.34% | (34 / 47) |
| parsing\_grammar.js |  | 91.59% | (98 / 107) | 56.25% | (9 / 16) | 96.55% | (28 / 29) | 91.59% | (98 / 107) |
| parsing\_operators.js |  | 72.12% | (150 / 208) | 65% | (52 / 80) | 70.45% | (31 / 44) | 72.12% | (150 / 208) |
| parsing\_translator.js |  | 79.7% | (161 / 202) | 71.43% | (165 / 231) | 87.5% | (28 / 32) | 79.7% | (161 / 202) |
| sugarpak.js |  | 96.62% | (143 / 148) | 82.86% | (58 / 70) | 100% | (30 / 30) | 96.58% | (141 / 146) |
| time\_period.js |  | 33.33% | (21 / 63) | 2.7% | (1 / 37) | 36.36% | (4 / 11) | 33.33% | (21 / 63) |
| time\_span.js |  | 20.65% | (19 / 92) | 1.72% | (1 / 58) | 16% | (4 / 25) | 20.65% | (19 / 92) |

Generated by istanbul at Sun Nov 16 2014 12:08:33 GMT-0800 (PST)
